# Can Exposure to Environmental Chemicals Increase the Risk of Diabetes Type 1 Development?

**DOI:** 10.1155/2015/208947

**Published:** 2015-03-26

**Authors:** Johanna Bodin, Lars Christian Stene, Unni Cecilie Nygaard

**Affiliations:** ^1^Department of Food, Water and Cosmetics, Division of Environmental Medicine, Norwegian Institute of Public Health, P.O. Box 4404, Nydalen, 0403 Oslo, Norway; ^2^Department of Chronic Diseases, Division of Epidemiology, Norwegian Institute of Public Health, P.O. Box 4404, Nydalen, 0403 Oslo, Norway

## Abstract

Type 1 diabetes mellitus (T1DM) is an autoimmune disease, where destruction of beta-cells causes insulin deficiency. The incidence of T1DM has increased in the last decades and cannot entirely be explained by genetic predisposition. Several environmental factors are suggested to promote T1DM, like early childhood enteroviral infections and nutritional factors, but the evidence is inconclusive. Prenatal and early life exposure to environmental pollutants like phthalates, bisphenol A, perfluorinated compounds, PCBs, dioxins, toxicants, and air pollutants can have negative effects on the developing immune system, resulting in asthma-like symptoms and increased susceptibility to childhood infections. In this review the associations between environmental chemical exposure and T1DM development is summarized. Although information on environmental chemicals as possible triggers for T1DM is sparse, we conclude that it is plausible that environmental chemicals can contribute to T1DM development via impaired pancreatic beta-cell and immune-cell functions and immunomodulation. Several environmental factors and chemicals could act together to trigger T1DM development in genetically susceptible individuals, possibly via hormonal or epigenetic alterations. Further observational T1DM cohort studies and animal exposure experiments are encouraged.

## 1. Introduction

Type 1 diabetes mellitus (T1DM) is an autoimmune disease with beta-cell destruction, resulting in insulin deficiency. A genetic predisposition seems to be necessary for developing the disease and is most often linked to genes in the HLA-complex [1]. About 90% of children with T1DM have the DR4-DQ8 haplotype and/or DR3-DQ2, and those who have both in combinations have the highest risk for T1DM. Islet autoantibodies are detected in ~90% of individuals at the time of diagnosis of T1DM, and these are directed against pancreatic proteins like insulin, glutamic acid decarboxylase (GAD), islet antigen 2 (IA-2), or zinc transporter 8 [2]. These autoantibodies generally appear in the circulation months to years before clinical onset.

It has become clear that environmental factors likely play a role in disease development, due to the facts that there has been an increasing incidence of type 1 diabetes in the last decades in many industrial countries and that there is less than 60% concordance of T1DM among monozygotic twins [3]. Factors like maternal age at delivery, infections in early life, deficiency of specific nutrients during pregnancy, and/or early childhood have been associated with risk of type 1 diabetes in observational studies [4, 5]. Other suggested environmental risk factors for T1DM are alterations in gut microbiota [6] and lack of general exposure to microbial factors (the “hygiene hypothesis”) [7].

**Figure 1:**
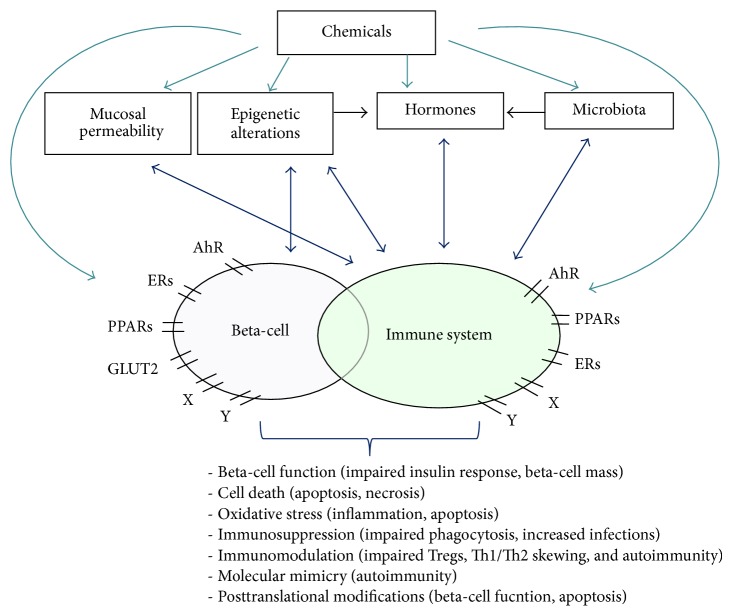
Mechanisms suggested to be involved in pathways of T1DM development after exposure to environmental chemicals via food/gut, air/lungs, and skin. Chemicals can act directly on beta or immune cells, by binding to receptors (X and Y-receptors could, for instance, be adrenergic-, purinergic-, or scavenger receptors) or after uptake in the cells by pinocytosis, endocytosis, or diffusion. Chemicals can also affect factors like mucosal permeability, the microbiome, or the hormone balance, all shown to interact with the immune system. Several chemicals have been shown to induce epigenetic changes. Chemical exposures can further lead to apoptosis or cell death, increased oxidative stress, impaired insulin response, altered immune function or immunosuppression, molecular mimicry, and posttranslational modifications.

This review will focus on the possible impact of environmental chemicals on T1DM development. The observed associations are summarized in Table 1 and the suggested mechanisms are summarized in Figure 1. Where little or no data of direct relevance for T1DM are available, we briefly discuss relevant data on other immune mediated diseases or on T2DM.

We start with a brief overview of relevant study designs and models, before we discuss relevant studies with the specific environmental chemicals, followed by a review of some potential mechanisms involved.

## 2. Study Designs Used in T1DM Research

The possible impact of environmental risk factors on T1DM development has been analysed in epidemiological studies by comparing the serum or urine levels of the chemical or their metabolites/biomarkers in T1DM patients and healthy controls. Interpretation of such studies must take into account the possibility that exposure may have occurred at or after diagnosis, by considering kinetics of the biomarkers and the ability of biomarkers to reflect actual exposure. Potential risk factors that could induce islet autoimmunity (presence of islet autoantibodies) or the progression from autoimmunity to development of disease can be investigated in longitudinal epidemiological studies with serial serum samples available from early childhood (from before the presence of autoantibodies and after seroconversion) up to diagnosis of T1DM. To our knowledge, chemicals have been determined in few prospective studies of type 1 diabetes in humans. Epidemiological studies have been limited by the low incidence of T1DM and the difficulty in accurately assessing exposures in epidemiological studies of large size.

Some epidemiological studies use proxies for environmental chemical exposures (for instance by self-report in questionnaires) or use ecological study designs where exposure is not determined at the individual level but only by region or country, and these must be interpreted with caution.

Animal models allow for controlled exposures of the chemical in question and are important for establishing causal relationships and the mechanistic mode of action. The most commonly used models for T1DM development are the nonobese diabetic (NOD) mouse and the Bio Breeding (BB) rat demonstrating spontaneous insulitis, influx of autoimmune cells into pancreatic islets attacking insulin producing beta-cells, and T1DM development [8–10]. Another frequently used model to induce diabetes in mice is by multiple low dose administrations of the beta-cell specific toxin streptozotocin, where the beta-cells are destroyed and the animals rapidly develop diabetes [11]. Increased levels of serum glucose and insulitis in pancreatic sections are examples of T1DM features in both animal models, whereas hyperinsulinemia and insulin resistance in other animal strains generally are signs of type 2 diabetes (T2DM) development. Other models of T1DM, including knock-out variants of the ones mentioned above, are reviewed elsewhere [12].

*In vitro* models are suitable to investigate direct effects on specific cell types, including receptor interactions. The most commonly used* in vitro* beta-cell systems for diabetes research are the rat beta-cell lines (INS-1E, RIN-m5F) [13, 14], the mouse beta-cell line (MIN6) [15] and primary islets and single beta-cells isolated from human, mouse, and rat pancreas. Decreased glucose secretion and increased apoptotic signaling are examples of T1DM-related mechanisms in beta-cells.

## 3. Environmental Chemicals

This review focuses on environmental chemicals that (i) have been found to contaminate food, water, and air and (ii) have been reported to influence the function of beta-cells or the immune system. These components include persistent organic pollutants (POPs like PCBs, dioxins, pesticides, and flame retardants), endocrine disruptors (bisphenol A, phthalates, and triclosan), certain metals (arsenic, organic derivatives of tin), N-nitroso compounds, bacterial toxins, ambient air pollution (such as ozone, particulate matter, and polycyclic aromatic hydrocarbons), and tobacco smoke. Some of these are persistent organic pollutants, which are resistant to environmental degradation and therefore accumulate in nature and the food chain. Other chemicals, including many of the endocrine disruptors (such as bisphenol A and phthalates) have a short half-life in the environment and have low bioaccumulation in humans.

### 3.1. Polychlorinated Biphenyls (PCBs)

There are 209 configurations of organochlorides with 1 to 10 chlorine atoms, classified as persistent organic pollutants. PCBs have been used as dielectric and coolant fluids in electrical equipment and can be found in marine food and wild animals due to accumulation in fatty tissue in the food chain.

In a prospective study on pregnant women with diabetes (primarily type 1), PCB serum levels were associated with the disease [16]. Another epidemiological study, however, showed tendency of an inverse association between maternal serum levels of PCB-135 or p,p′-DDE during pregnancy and T1DM development in the child, but this was not statistically significant [17]. In support of PCB effects on autoimmunity, employees working at a PCB production factory had higher prevalence of antiglutamic acid decarboxylase (anti-GAD) autoantibodies in their serum compared to controls [18].

Animal studies reveal induced insulin resistance, indicating T2DM development, after exposure to a mixture of persistent organic pollutants that mimics the relative abundance of organic pollutants present in crude salmon oil [19, 20], and there are several studies indicating associations between serum PCB levels and T2DM in humans [21, 22]. Dioxins and dioxin like PCBs act via the aryl hydrocarbon receptor (AhR) and can cause oxidative stress, apoptosis, and increased inflammation during metabolization/detoxification of the chemical [23].

### 3.2. 2,3,7,8-Tetrachlorodibenzo-p-dioxin (TCDD)

TCDD is primarily formed as a byproduct in the manufacturing of materials requiring the use of chlorinated phenols and during the combustion of chlorinated chemical products. TCDD is a persistent organic pollutant that has been used in herbicides like Agent Orange [24]. Humans are mostly exposed through intake of marine food and game due to accumulation of the chemical in fatty tissue in the food chain.

There are no epidemiological studies investigating associations between TCDD exposure and T1DM. However, TCDD has been shown to be highly toxic for INS-1E rat pancreatic beta-cells regarding survival and ultrastructure via activation of the aryl hydrocarbon receptor (AhR) [25]. Further, experimental studies have shown that TCDD exposure in C57BL/6J mice impaired glucose-stimulated secretion of insulin from the islets via the AhR signaling pathway [26]. TCDD has also been shown to induce calcium influx via T-type channels, regulating vesicular trafficking, such as lysosomal and secretory granule exocytosis, indicating that TCDD might exert adverse effects on beta-cells by stimulating continuous insulin release resulting in beta-cell exhaustion in an INS-1 rat beta-cell line [27].

On the other hand, in the NOD mouse model, TCDD has been shown to prevent T1DM development when administered from 8 weeks of age, a time point after the spontaneous insulitis development is normally initiated (starting from 4 weeks of age in the NOD mice [28]), due to increased number of regulatory T-cells in the pancreas and reduced insulitis [29]. The immunosuppressive effect of TCDD has been shown to be due to activation of the aryl hydrocarbon receptor (AhR), which is a ligand-activated transcription factor in CD4+ Th17 T-cells, and upregulation of IL-22 expression [30]. IL-22 is secreted by Th17 cells and is highly present in various autoimmune diseases, but whether IL-22 is mediating the inflammation itself, or is a byproduct of the inflammation is depending on the tissue and overall cytokine setting [31]. In agreement with this, in another murine model for autoimmunity, systemic lupus erythematosus (SLE), TCDD appears to promote differentiation of regulatory T-cells via AhR and inhibiting Th17 cells and cause immunosuppressive effects [32, 33]. Both models indicate a therapeutic effect of AhR activation in autoimmunity development in adult animals. In line with the immunosuppressive effects of TCDD via Tregs stimulation following AhR ligation/activation, TCDD has also demonstrated suppressive (preventive) effects in rodent allergy models [34, 35]. Interestingly, other AhR ligands did not have this suppressive effect on the allergy development, suggesting that the effect via AhR is ligand specific [34]. On the other hand, TCDD administered during gestation induced adult autoimmunity in different mice strains [36–38], suggesting that exposure to chemicals during critical developmental stages* in utero* may possibly promote the development of autoimmune diseases, including T1DM, later in life.

Human serum levels of TCDD have been associated with increased insulin plasma levels and T2DM, although there are some conflicting results from epidemiological studies [39–42].

### 3.3. Dichlorodiphenyltrichloroethane (DDT) and Dichlorodiphenyldichloroethylene (DDE)

The organochloride DDT and its metabolite DDE have been used as insecticides triggering spasms via the opening of neuronal ion channels and are persistent chemicals that accumulate in fatty tissue in the food chain.

In a nested case-control study maternal serum levels of p,p′-DDE during pregnancy and T1DM development in the child, there was no significant association with T1DM; however the T1DM cases had a tendency of lower p,p′-DDE levels than control subjects (as mentioned above for PCB) [17].

Serum levels of DDT and DDE are both associated with the development of T2DM [33, 43, 45–47]. It has been shown that DTT activates AhR-signaling and can induce apoptosis in murine embryonic neuronal cells, but there are no reports available about beta-cell toxicity [146].

### 3.4. Polybrominated Diphenyl Ethers (PDBE)

PDBEs are bioaccumulating persistent chemicals used as flame retardants in building materials, textiles, furnishings, and electronics. Exposure to humans is mainly via ingestion of food and by inhalation of indoor air and they can act as an endocrine disruptors.

There are no epidemiological studies investigating associations between PDBE exposure and T1DM in humans. In a rat exposure study 2,2′,3,3′,4,4′,5,5′,6,6′-decabromodiphenyl ether (BDE209) exposure was shown to induce hyperglycemia, decrease insulin, glutathione, and superoxide dismutase serum levels and increase TNF*α* serum levels, probably via induction of oxidative damage and was further correlated to changes in rat liver cell MHC and TNF*α* transcripts that possibly could be involved in T1DM development [49]. In a study on porcine alveolar cells, a mix of PDBE, DE-71 was shown to induce lower levels of proinflammatory cytokine release compared to control, indicating that PDBEs may suppress innate immunity [50].

PDBEs have been suggested to be associated with altered thyroxin hormone levels, and there are conflicting reports on association with T2DM in humans [47, 48, 51, 52].

### 3.5. Perfluorinated Alkyl Substances (PFAS)

PFAS have attractive lipid and water repelling properties and are therefore used in fire-fighting foam, textiles, kitchen ware, and food packaging materials. Human exposure to PFAS is mainly through diet via marine food and game.

An epidemiological study has reported that increased serum level of the perfluorononanoic acid (PFNA) in human adolescents is associated with decreased blood insulin and beta-cell function [54].

PFAS exposure* in utero* appears to modulate the immune response in children, resulting in reduced immune responses to vaccines and increased infections in early childhood [55, 57]. In a human cumulative health risk assessment report PFAS are suggested to be immunotoxic, although the mechanisms are unknown and possible multiple [58]. Further, elevated PFNA serum level was also associated with diabetes in an elderly population supporting the view that PFAS can alter glucose metabolism in humans and induce T2DM [53]. Lv et al. [56] reported that PFAS exposure in rats during gestation and lactation altered glucose tolerance in adult offspring.

### 3.6. Bisphenol A (BPA)

BPA is used in the production of polycarbonate plastic and epoxy resins coating the inside of metal cans and can leak from the plastic into food. Human exposure is ubiquitous, as BPA metabolites are measured in more than 90% of children and adults in westernized countries [147]. BPA is rapidly metabolized and more than 99% is secreted in the urine within 4 hours [148], making detection in human blood samples variable and inconsistent at the limit of detection.

No human study of BPA exposure and T1DM development has been performed. Using the NOD mouse model, BPA was found to increase the spontaneous T1DM development after both long term postnatal exposure and short term prenatal and early life exposure [59, 60]. A very high BPA exposure (resembling 15 mg/kg/day) showed tendency to a preventive effect, which possibly could be explained by different mechanisms dominating at higher BPA exposure, such as an increased insulin secretion or estrogenic compensation mechanisms. These studies suggest that BPA acts by impairing macrophage function, resulting in impaired clearance of apoptotic cells, a feature common for several autoimmune diseases. BPA was also seen to modulate immune responses in lymphoid tissue in the mice and to impair islet morphology and beta-cell function in isolated rat pancreatic islets [60, 62].

Epidemiological studies have shown both positive and no associations to T2DM [61, 63, 65, 67–69]. In addition, BPA exposure has also been associated with asthma development in both human epidemiological studies and animal experimental studies [149–152]. It has been shown that BPA induces insulin secretion, in both human and mouse beta-cells via ER*β* activation, possibly contributing to T2DM development [64, 66]. Further animal studies have shown induced insulin resistance and T2DM in mice [66, 145, 153–157].

### 3.7. Triclosan

Triclosan is a chlorinated aromatic compound that has anti-inflammatory effects, suppressing microbial-pathogen recognition pathway molecules and chronic mediators of inflammation and is used as antimicrobial agent in soap, toothpaste, clothes, and suture material for medical surgery [158].

There are no epidemiological studies investigating associations between triclosan exposure and T1DM or T2DM; however, triclosan exposure has been associated with increased risk of sensitization, rhinitis, and food allergy [159–161]. As an endocrine disruptor, triclosan has been shown to decrease thyroid hormone levels in humans and in rats [71–73]. Treatment with tri-iodothyronine (T3) in the BB rat reduced T1DM incidence and increased beta-cell mass in diabetes free Wistar rats [162], indicating that modulation of thyroid hormone levels may affect T1DM development in genetically susceptible animals.

### 3.8. Phthalates

Phthalates are commonly used as plasticizers and in a variety of consumer products, like paint and cosmetics. Phthalates are rapidly biodegradable endocrine disruptors and human exposure is mainly through diet via contamination from plastic into food and via inhalation of phthalates in dust in indoor air. Uptake via the skin from cosmetic products is also contributing to systemic exposure.

No epidemiological studies have so far investigated associations between phthalate exposure and T1DM. There are, however, epidemiological studies showing associations with phthalate exposure and insulin resistance and T2DM [62, 74, 76, 78, 80–83]. Phthalates can induce sustained oxidative stress and inflammation via activation of AhR, ER, and/or binding to peroxisome proliferator activated receptor PPARs [75, 77, 79, 163, 164]. Phthalate exposure is also associated with asthma development and Th2 deviation, possibly via epigenetic modulations [164–167].

### 3.9. Arsenic

Arsenic is often contaminating drinking water from private wells, in high levels especially throughout South East-Asia and Latin-America but also in lower levels in parts of the United States, Australia, and Europe [86, 168].

There are no epidemiological studies investigating associations between arsenic exposure and T1DM development. Arsenic exposure has been shown to impair the immune system in humans and animal models [86, 89, 91] and to alter the gut microbiome diversity, microbiome metabolic profiles as well as inhibiting the glucose stimulated insulin release in mice [85, 93]. Further, prenatal arsenic exposure has been associated with increased miRNAs (miR-107 and miR-126) involved in signaling pathways related to diabetes [84, 169] and another possible mechanism for diabetes development can be a direct negative effect on beta-cell functions and apoptosis due to arsenic exposure seen in MIN6 pancreatic murine and RIN-m5F rat beta-cell lines [44, 88].

Epidemiological studies have reported an association between arsenic exposure and T2DM [87, 90, 92, 94, 170].

### 3.10. Organotin Compounds

Organotin compounds are used as stabilizers in the production of polyvinyl chloride, and triphenyltin compounds are used as antifungal agents [171].

There are no present epidemiological study investigating associations between organotin compounds and diabetes in humans. However, triphenyltin exposure has been shown to cause hyperglycemia in rabbits and hamsters, possibly due to inhibitory effects on insulin secretion by decreasing the glucose-induced rise in intracellular Ca^2+^ in pancreatic beta-cells, as shown in triphenyltin exposed hamsters [95, 96]. Triphenyltin exposure did not affect diabetes development in rats and mice [98], although it has recently been shown that tributyltin chloride induces pancreatic islet cell apoptosis in male KM mice [97].

### 3.11. N-Nitroso Compounds

N-nitroso compounds are present in processed food [172] but can also be formed in the gastrointestinal tract when nitrates from food or water are converted to nitrites and reacts with amines. These compounds are shown to be toxic to pancreatic beta-cells [102].

Higher levels of nitrates in drinking water have been associated with increased incidence of T1DM [105–107], although case-control studies on children's dietary intake of nitrates show conflicting results [99–101, 103, 104, 173–178]. Nitrosamines in food additives have been associated with a higher risk for T1DM development [99–101].

An exposure study with smoked/cured mutton containing N-nitroso compounds, fed at the time of mating, during gestation and in early life in the normal nondiabetic mouse strain CD1 showed development of diabetes in the offspring, more pronounced in male offspring compared to females (16% compared to 4%) [100].

### 3.12. Vacor

N-nitroso-compounds have previously been used as pest control chemicals and the rodenticide Vacor is shown to specifically decrease beta-cell functions by inhibiting mitochondrial ATP production and suppressed glucose-induced insulin secretion in isolated rat pancreatic islets and beta-cells [116, 118–120].

### 3.13. **Streptozotocin**

Streptozotocin is a naturally occurring glucosamine-nitrosourea compound produced by the soil microbe* Streptomyces achromogenes* causing destruction of beta-cells via DNA fragmentation, activating poly ADP-ribosylation, formation of superoxide radicals, hydrogen peroxide, and liberation of nitric oxide [11, 108, 113]. It is exclusively taken up by beta-cells via the glucose transport protein GLUT2 due to its similarity to glucose and the toxicity is therefore specific to beta-cells [109, 111]. Multiple low dose exposure of streptozotocin is used in animal studies to induce beta-cell destruction associated with pancreatic insulitis and subsequent T1DM-like symptoms [108, 110, 112]. Another toxic glucose analogue used in rodents to induce diabetes is alloxan, an oxygenated pyrimidine derivative. Alloxan generates reactive oxygen species (ROS), superoxide radicals, hydrogen peroxide, and, in a final iron-catalysed reaction step, hydroxyl radicals that together with increased cytosolic calcium concentration induce beta-cell death [11, 108, 114].

### 3.14. Bafilomycin

Bafilomycin from* Streptomyces*-infected vegetables has been shown to specifically decrease beta-cell function, seen as reduction of islet size and beta-cell mass after injection in mice [116, 179]. Bafilomycin exposure* in utero*, but not after birth, significantly accelerated the onset and incidence of diabetes in NOD mice [115], indicating that naturally occurring environmental toxicants possibly could influence T1DM risk. However, such association has not been investigated in epidemiological T1DM studies. Furthermore, high dose bafilomycin exposure was shown to promote cell death whereas low dose induced insulin secretion in the MIN6 mouse pancreatic cell line [117].

### 3.15. Cereulide

Cereulide is a toxin produced by certain strains of* Bacillus cereus*, a bacterium connected to emetic food poisonings from raw milk and industrially produced baby food [180].

There are no epidemiological studies investigating associations between cereulide exposure in human and diabetes development.

Cereulide has, however, been shown to cause necrotic cell death in porcine pancreatic Langerhans islets in cell culture [121] and to induce mitochondrial stress markers (p53 upregulated modulator of apoptosis, Puma, and CCAAT/-enhancer-binding protein homologous protein, CHOP) and apoptosis in mouse (MIN6) and rat (INS-1E) beta-cell lines, as well as in mouse islets [122].

### 3.16. Air Pollution

Cumulative exposure to ozone and sulphate in ambient air in Southern California has been associated with T1DM development [129]. Animal ozone exposure experiments, however, revealed induced glucose intolerance in rats [130]. Further, carbon monoxide (CO) has been associated with T2DM [131, 132]. Interestingly, carbon monoxide has been used as treatment of T1DM in the NOD mouse model due to its anti-inflammatory and antiapoptotic properties [133].

Exposure to particulate matter (PM) induces formation of reactive oxygen species in human lung endothelial cells and circulating monocytes, leading to DNA damage and inflammation [124, 126].

Fine particulate matter (PM2.5) has been associated with diabetes in rats, by intratracheal instillation, enhanced insulin resistance, and visceral inflammation in rats fed a high fat diet but not a normal chow [70, 128]. In humans, air pollution measured as outdoor PM < 10 *μ*m in aerodynamic diameter (PM10) and nitrogen dioxide (NO2) has been shown to be associated with T2DM [123, 125] and decreased insulin sensitivity [127].

### 3.17. Tobacco Smoke

Maternal smoking during pregnancy has been associated with decreased T1DM development [135, 136, 138–142], although passive smoking was more frequent in children with T1DM in one study [129].

An association between prenatal and postnatal tobacco smoke and increased insulin resistance has been shown in 10 year old children [134]. Further studies have shown increased risk of T2DM due to maternal smoking, as well as increased insulin resistance and increased risk of T2DM development due to direct smoking in adults [137].

### 3.18. Polycyclic Aromatic Hydrocarbons (PAHs)

PAHs are found in fossil fuels and tar deposits and are produced during incomplete combustion of organic matter and thus are abundant in air pollution. In addition, considerable PAH exposure is experienced from dietary sources [181].

We are not aware of any studies on PAH and risk of T1DM in humans. Animal and human* in vitro* cell studies link PAH exposure to the generation of oxidative stress, DNA damage and inflammation via activation of the aryl hydrocarbon receptor (AhR) in the metabolism and secretion of the PAHs by CYP enzymes [23, 124, 126, 182].

The impaired regulatory T cell (Treg) function associated with human PAH and ambient air pollution exposure, explained by increased methylation of the transcription factor Foxp3 in Tregs [144], may be a plausible mechanism for promoting T1DM development, although this has not yet been investigated. Epidemiological studies have shown association between urinary PAH levels and T2DM development [143].

## 4. Mechanisms for Chemical-Induced Triggering of T1DM

### 4.1. Toxic Effects on Beta-Cells

Direct effects on beta-cell function or viability could be a mechanism of environmental chemical for contributing to autoimmunity. Suggested mechanisms leading to beta-cell apoptosis are related to altered mitochondrial functions and induction of oxidative stress. Other mechanisms than apoptosis leading to reduced beta cell mass include impairment of beta-cell replication, by cAMP suppression via *α*_2_-adrenergic receptors and thereby reducing total beta-cell mass [183]. It has also been shown that adenosine receptor agonists acting through the adenosine receptor A2aa, increased beta-cell proliferation and accelerated restoration of normoglycemia in zebrafish [184]. Regarding ATP purinoceptors, increased beta-cell apoptosis has been reported in P2X(7) knock-out mice [185]. Glucose is shown to induce ATP release in a mouse beta-cell line and ADP activation of P2Y(13) receptors to inhibit insulin release [186]. In rodent as well as human pancreatic beta-cells, extracellular ATP has been proposed as a paracrine signal amplifying glucose-induced insulin secretion via P2X(3) receptor activation [187]. Further, it has been reported that ATP activation of P2X(7) receptors in peritoneal mouse macrophages mediated free fatty acid release, substrate for many enzymes including cyclooxygenases that promote inflammation [188]. Environmental chemicals could possibly induce extracellular accumulation of ATP following Th2-type inflammatory responses, similar to what has been shown for airborne fungal allergens in naïve mice [189]. Activation of estrogen receptors ER*α* can cause enhancement of glucose-induced insulin biosynthesis, reduction in islet toxic lipid accumulation and promote beta-cell survival from pro-apoptotic stimuli, and activation of ER*β* can increase glucose induced insulin secretion in both rodent and human beta-cells [190]. Activation of AhR can induce oxidative stress, DNA damage and inflammation [23]. Chemicals influencing the gap junctions between beta-cells could increase toxicity and susceptibility to cytokine induced apoptosis, as shown when downregulating connexin36 in INS1E-cells, suggested to be involved in Ca^2+^ homeostasis within the endoplasmatic reticulum ER [191].

BPA, PFAS, TCDD, streptomycin, alloxan, N-nitroso compounds, streptozotocin, zinc, organotins, and bafilomycin are all shown to cause alterations in beta-cell function and structure and/or apoptosis in animal studies [44, 54, 59, 60, 62, 88, 102].

### 4.2. Immunomodulation

In addition to direct effects on beta-cell numbers and function and glucose-insulin balance, environmental chemicals may affect T1DM development by modulating the function of innate and adaptive immune cells. Recurring infections in early childhood could trigger the immune system and possibly boost autoimmunity, and enteroviral infections in early life are associated with T1DM development [192, 193]. As an example PFAS exposure* in utero* appears to modulate the immune response in children, resulting in reduced vaccine responses and increased infections in early childhood [55, 57]. The increased risk of infections may indirectly give increased risk of enteroviral infections triggering T1DM development in children with auto antibody positivity. Other chemicals, such as for instance PAHs, are reported to reduce the numbers and function of regulatory T-cells [144, 194], cells that are important in the suppression of the autoreactive T-cells that are key players in the induction of autoimmunity. Corsini et al. [195] showed that several PFAS decrease LPS-induced cytokine secretion in human peripheral blood leucocytes and Brieger et al. [196] showed a direct increased cytotoxicity to human NK cells by PFAS (PFOA) exposure. Altered cytokine secretion, reduced regulatory T-cells or Th17 cells by environmental chemicals could be plausible explanations for a modified immune response and the development of autoimmunity. It has been shown that exposure not only to PFAS, but also to BPA, phthalates, arsenic, PCB, and air pollution can alter the cytokine balance in human and mice cells* in vitro* and a shift in cytokine balance is further associated with development of autoimmunity [59, 60, 195, 197–202].

Another suggested immunological process linking viral infections to T1DM onset is molecular mimicry [203]. There is a high degree of homology between human Glutamic Acid Decarboxylase GAD65, a pancreatic enzyme considered to be an important autoantigen involved in T1DM development, and a heat shock protein from the* Mycobacterium avium subspecies paratuberculosis*, MAP Hsp65, and it has been shown that T1DM patients can have antibodies against MAP Hsp65 [204]. It has been suggested that there is a cross-reactivity between MAP Hsp65 and GAD65, implying that biological mimicry potentially could be a mechanism of triggering TIDM. It has also been reported that antibodies from T1DM patients recognizing MAP3865c epitopes from the* Mycobacterium avium* could cross-react with ZnT8, another autoantigen in T1DM development [205], although this hypothesis has not been verified in epidemiological studies. Other environmental factors, including chemicals, can in principal change exogenous and endogenous proteins, leading to mimicry pathways of T1DM triggering.

Improper activation of the immune system may lead to allergy development or trigger autoimmunity, and exposure to PFAS, arsenic, BPA, phthalates, air pollution, ozone, nitric oxide, particulate matter, triclosan, PAHs, tobacco smoke, dioxin, and PCBs have all been reported to be associated with asthma and/or allergy in several epidemiological studies [42, 149, 150, 165, 206–215].

BPA and PDBEs have been shown to reduce cytokine secretion from macrophages, and BPA and arsenic seem to impair phagocytic activity in macrophages, possibly leading to a reduced clearance of apoptotic cells in pancreatic islets which can result in an induced insulitis in the NOD mouse [50, 91, 216].

### 4.3. Epigenetics

Epigenetic alterations, via histone modifications, DNA methylation and microRNA dysregulation leading to altered gene expression, represent one way in which chemicals can induce effects early in life that manifest disease later in life. Emerging data suggest that prenatal exposures, like for instance to arsenic, may induce epigenetic alterations, already measurable in umbilical cord blood [84, 217]. Prenatal exposure to phthalates and postnatal exposure to BPA have been suggested to work together in a “two-hit model” on hormonal alterations leading to epigenetic regulation of gene expression [149]. Phthalate exposure has been shown to induce DNA methylation of the estrogen receptor alpha in a breast cancer cell line [218]. Environmental factors such as pharmaceuticals, pesticides, air pollutants, industrial chemicals, heavy metals, hormones, nutrition, as well as behavior have been suggested to change gene expression with demonstrated changes in epigenetic markers [214, 218, 219]. Alterations in micro-RNA levels might influence beta-cell functions and overexpression of microRNA miR375 has been shown to be associated with suppressed glucose induced insulin secretion by reduced levels of PDK1 leading to reducing beta-cell viability and cell number [220]. IL-1*α* and TNF*α* induce miR21, miR34a, and miR146a in human and NOD mouse pancreatic islets and in the mouse MIN6 beta-cell line and are involved in cytokine-induced cell death [221]. The miR21 as well as miR34a reduces beta-cell apoptosis and protects against T1DM development [222, 223], while overexpression of miR29a/b/c was reported to promote beta-cell apoptosis [224].

### 4.4. Microbiota

The microbiota composition in the gut has been shown to be crucial for developing a healthy immune system in animals. The right composition is suggested to support oral tolerance and protect against enteral virus infections, and microbial colonization of* Bifidobacterium* has been shown to be lower in patients with T1DM [6, 225–230]. Transfer of microbiota from Myd88-/-NOD mice, which are protected from diabetes, has been shown to reduce insulitis and delay T1DM development in the normal diabetes prone NOD recipient [231]. Furthermore, alterations in microbiota composition results in altered hormone levels in the NOD mouse [232]. Nutritional and chemical constituents in our diet and drinking water have been shown to alter the microbiota composition in animals [233–235] and future studies are needed to clarify the importance of such interactions between environment, microbial flora and autoimmunity. On the other hand, probiotics could possibly interfere with T1DM development and examples hereof are animal studies with probiotics given to the T1DM prone NOD mouse showing protective effects against T1DM development via Th17 induction [236–239]. In an epidemiological context, the ongoing PRODIA study will elucidate if introduction to probiotics during the first 6 months of life decreases the appearance of T1DM-associated autoantibodies in children with genetic risk for T1DM [240].

### 4.5. Intestinal Permeability

Increased intestinal permeability is an early feature of diabetes before the onset of the disease in the Bio Breeding T1DM rat model, and blocking of the tight junction modulator zonulin has been shown to inhibit the disease in this model [241, 242]. Increased intestinal permeability has also been shown to be an early event in T1DM patients with upregulation of zonulin prior to the onset of the disease [229, 243–247].

Chemicals, like heavy metals and organochloride pesticides, can possibly affect intestinal permeability, as well as impairing the osmoregulation and calcium transport [248, 249].* Lactobacillus* has been shown to reduce the intestinal permeability via relocation of occludin and ZO-1 into the tight junction area between duodenal epithelial cells after short term administration to healthy volunteers [250] and this mechanism together with alterations in hormone levels could possibly explain a beneficial effect of probiotics in the NOD mouse model [236–239].

## 5. Summary

We have presented literature supporting a possible role of environmental chemicals to act as triggers or accelerators for T1DM development. Chemicals may have direct toxic effects on insulin producing beta-cells or have immune modulatory effects, alter hormone levels, affect the microbiota, or alter intestinal permeability. Chemical-induced epigenetic alterations leading to altered gene expression are probably involved, in particular in relation to* in utero* effects.

Whether the doses of environmental chemicals to which humans are exposed are sufficient to impact the risk of T1DM remains largely unexplored. Due to lack of strong evidence for a single factor as the major trigger for T1DM development it is tempting to propose that several factors have additive or synergistic effects, acting via several mechanisms and/or at different stages in the disease development. Human exposure to environmental chemicals is complex. While some chemicals may have beneficial effects, others may have detrimental effects in individuals with autoimmune predisposition, and the adverse consequences of this sum of exposures cannot be elucidated with the information available. Further observational T1DM cohort studies with determination of several biomarkers of chemical exposure in serum and urine, together with animal and cellular experiments using single and combined chemical exposures are encouraged.

## Figures and Tables

**Table 1:** Summary of studies reporting associations between exposure to environmental chemicals and endpoints relevant to T1DM development (T1DM, T2DM or beta-cell/immunomodulations). A “+” sign in front of the reference indicates positive association between chemical exposure and the respective endpoint (diabetes or beta-cell/immune modulations), while a “−” sign indicates no association between chemical exposure and T1DM or having inverse associations. “(+)” in front of the reference indicates that the exposure was measured in common drinking water, not for the individual, resulting in more uncertain conclusions.

Subgroup of chemicals	Environmental chemical	TIDM epidemiology	TIDM animal studies	T2DM epidemiology	Beta-cell modulations	Immuno modulations	Likelihood for T1DM influence
Polychlorinated biphenyls	PCB	+ Longnecker et al. 2001 [16]		+ Everett et al. 2011 [22]		+ Schmidt and Bradfield 1996 [23]	+++ (−)
		+ Langer et al. 2002 [18]		+ Carpenter 2006 [21]			
		− Rignell-Hydbom et al. 2010 [17]					
Dioxins	TCDD/dioxin		− Shinomiya et al. 2000 [28]	+ Cranmer et al. 2000 [39]	+ Martino et al. 2013 [25]	− Rohlman et al. 2012 [30]	++ (−)
			− Kerkvliet et al. 2009 [29]	+ Pelclová et al. 2006 [40]	+ Kurita et al. 2009 [26]	− Li and McMurray 2009 [33] − Schulz et al. 2012a, b [34, 35]	
				− Warner et al. 2013 [41]	+ Kim et al. 2009 [27]	− Hanieh 2014 [32]	
						+ Ishimaru et al. 2009 [36]	
						+ Mustafa et al. 2011a, b [37, 38]	
Organochlorides (pesticides)	DDT/DDE	− Rignell-Hydbom et al. 2010 [17]		+ Codru et al. 2007 [43]		+ Yang et al. 2012 [44]	++ (−)
				+ Philibert et al. 2009 [45]		− Li and McMurray 2009 [33]	
				+ Taylor et al. 2013 [46]			
				+ Turyk et al. 2009 [47]			
Polybrominated biphenyls (Flame retardants)	PDBE			+ Lee et al. 2011 [48]	+ Zhang et al. 2013 [49]	+ Hennigar et al. 2012 [50]	++
				− Turyk et al. 2009 [47]		+ Turyk et al. 2008 [51]	
				+ Llm et al. 2008 [52]			
Perfluorinated alkyl substances	PFAS			+ Lind et al. 2014 [53]	+ Lin et al. 2009 [54]	+ Grandjean et al. 2012 [55]	++
					+ Lv et al. 2013 [56]	+ Granum et al. 2013 [57]	
						+ Borg et al. 2013 [58]	

Endocrine disruptors	BPA		+ Bodin et al. 2013 [59]; Bodin et al. 2014 [60]	+ Aekplakorn et al. 2014 [61]	+ Song et al. 2012 [62]	+ Bodin et al. 2014 [60]	++++
				+ Ahmadkhaniha et al. 2014 [63]	+ Soriano et al. 2012 [64]		
				− Kim and Park 2013 [65]	+ Nadal et al. 2009 [66]		
				+ Sabanayagam et al. 2013 [67]			
				+ Shankar and Teppala 2011 [68]			
				+ Silver et al. 2011 [69]			
				+ Sun et al. 2014 [70]			
	Triclosan					+ Paul et al. 2009 [71]	+
						+ Zorrilla et al. 2009 [72]	
						+ Koeppe et al. 2013 [73]	
	Phthalates			+ Huang et al. 2014 [74]		+ Mankidy et al. 2013 [75]	+
				+ James-Todd et al. 2012 [76]		+ Vetrano et al. 2010 [77]	
				+ Kim et al. 2013 [78]		+ Sarath Josh et al. 2014 [79]	
				+ Lind et al. 2012 [80]			
				+ Stahlhut et al. 2007 [81]			
				+ Svensson et al. 2011 [82]			
				+ Trasande et al. 2013 [83]			

Metals	Arsenic			+ Rager et al. 2014 [84]	+ Douillet et al. 2013 [85]	+ Dangleben et al. 2013 [86]	++
				+ Tsai et al. 1999 [87]	+ Lu et al. 2011 [88]	+ Ahmed et al. 2012 [89]	
				+ Bräuner et al. 2014 [90]	+ Yang et al. 2012 [44]	+ Banerjee et al. 2009 [91]	
				+ Lee and Kim 2013 [92]		+ Lu et al. 2014 [93]	
				+ Mahram et al. 2013 [94]			
					
	Organotins				+ Miura et al. 1997 [95]; Miura et al. 2012 [96]		+
					+ Zuo et al. 2014 [97]		
					− Matsui et al. 1984 [98]		

Nitroso compounds	Nitrates and nitroso amines	+ Dahlquist et al. 1990 [99]			+ Helgason and Jonasson 1981 [100]		++ (+)
		+ Benson et al. 2010 [101]			+ Wilson et al. 1983 [102]		
		− Samuelsson et al. 2011 [103]					
		− Cherian et al. 2010 [104]					
		(+) Kostraba et al. 1992 [105]					
		(+) Parslow et al. 1997 [106]					
		(+) van Maanen et al. 1999 [107]					
		(+) Helgason 1991					
	Streptozotocin		+ Szkudelski 2001 [108]		+ Schnedl et al. 1994 [109]		++
			+ Leiter 1982 [110]		+ Wang and Gleichmann 1998 [111]		
			+ Rossini et al. 1977 [112]		+ Lenzen 2008 [11]		
	Alloxan		+ Rerup 1970 [113]		+ Lenzen 2008 [11]		++
					+ Szkudelski 2001 [108]		
					+ Eizirik et al. 1994 [114]		
	Bafilomycin		+ Hettiarachchi et al. 2004 [115]		+ Myers et al. 2003 [116]		++
					+ Hettiarachchi et al. 2006 [117]		
	Vacor				+ Myers et al. 2003 [116]		+
					+ Esposti et al. 1996 [118]		
					+ Taniguchi et al. 1989 [119]		
					+ Wilson and Gaines 1983 [120]		
	Cereulides				+ Virtanen et al. 2008 [121]		+
					+ Vangoitsenhoven et al. 2014 [122]		

Air pollution	Particulate matter			+ Eze et al. 2014 [123]		+ Danielsen et al. 2011 [124]	++
				+ Hathout et al. 2001 [125]		+ den Hartigh et al. 2010 [126]	
				+ Brook et al. 2013 [127]		+ Yan et al. 2011 [128]	
	Ozone	+ Hathout et al. 2006 [129]				+ Bass et al. 2013 [130]	+++
	Carbon monoxide			+ Janghorbani et al. 2014 [131]			+
				+ Dales et al. 2012 [132]			
				− Nikolic et al. 2014 [133]			
	Maternal/smoking	+ Hathout et al. 2006 [129]		+ Thiering et al. 2011 [134]	+ Rasouli et al. 2013 [135]		+++ (−)
		− Dahlquist and Kallen 1992 [136]		+ Persson et al. 2000 [137]			
		− Hjern and Söderström 2008 [138]					
		− Ievins et al. 2007 [139]					
		− Johansson et al. 2008 [140]					
		− Marshall et al. 2004 [141]					
		− Rasouli et al. 2013 [135]					
		− Robertson and Harrild 2010 [142]					
	PAH			+ Zhao et al. 2014 [183]		+ Nadeau et al. 2010 [144]	++
	polycyclic aromatic hydrocarbon					+ den Hartigh et al. 2010 [126]	
						+ Danielsen et al. 2011 [124]	
						+ Perreault et al. 2013 [145]	

^+^Beta-cell toxicity or immunomodulation.

^++^Beta-cell toxicity and immunomodulation.

^+++^Beta-cell toxicity or immunomodulation and T1DM human or animal study.

^++++^Beta-cell toxicity and immunomodulation and T1DM human or animal study.
